# Mechanochemically Synthetized PAN-Based Co-N-Doped Carbon Materials as Electrocatalyst for Oxygen Evolution Reaction

**DOI:** 10.3390/nano11020290

**Published:** 2021-01-22

**Authors:** Paulette Gómez-López, José Ángel Salatti-Dorado, Daily Rodríguez-Padrón, Manuel Cano, Clemente G. Alvarado-Beltrán, Alain R. Puente-Santiago, Juan J. Giner-Casares, Rafael Luque

**Affiliations:** 1 Grupo FQM-383, Departamento de Química Orgánica, Universidad de Córdoba, E-14071 Córdoba, Spain; z82golop@uco.es (P.G.-L.); dailydggs@gmail.com (D.R.-P.); 2 Departamento de Química Física y Termodinámica Aplicada, Instituto Universitario de Nanoquímica (IUNAN), Facultad de Ciencias, Universidad de Córdoba, Campus de Rabanales, Ed. Marie Curie, E-14071 Córdoba, Spain; a72sadoj@uco.es (J.Á.S.-D.); q82calum@uco.es (M.C.); 3 Facultad de Ingeniería Mochis, Universidad Autónoma de Sinaloa, Fuente de Poseidón y Prol. Angel Flores, S.N., 81223 Los Mochis Sin., Mexico; calvarado@uas.edu.mx; 4 Department of Chemistry and Biochemistry, University of Texas at El Paso, 500 West University Avenue, El Paso, TX 79968, USA; arpuentesan@utep.edu; 5 Scientific Center for Molecular Design and Synthesis of Innovative Compounds for the Medical Industry, People’s Friendship University of Russia (RUDN University), 117198 Moscow, Russia

**Keywords:** mechanochemical synthesis, carbon N-doped, Co_2_O_3_ nanoparticles, PAN, OER

## Abstract

We report a new class of polyacrylonitrile (PAN)-based Co-N-doped carbon materials that can act as suitable catalyst for oxygen evolution reactions (OER). Different Co loadings were mechanochemically added into post-consumed PAN fibers. Subsequently, the samples were treated at 300 °C under air (PAN-A) or nitrogen (PAN-N) atmosphere to promote simultaneously the Co_3_O_4_ species and PAN cyclization. The resulting electrocatalysts were fully characterized and analyzed by X-ray diffraction (XRD) and photoelectron spectroscopy (XPS), transmission (TEM) and scanning electron (SEM) microscopies, as well as nitrogen porosimetry. The catalytic performance of the Co-N-doped carbon nanomaterials were tested for OER in alkaline environments. Cobalt-doped PAN-A samples showed worse OER electrocatalytic performance than their homologous PAN-N ones. The PAN-N/3% Co catalyst exhibited the lowest OER overpotential (460 mV) among all the Co-N-doped carbon nanocomposites, reaching 10 mA/cm^2^. This work provides in-depth insights on the electrocatalytic performance of metal-doped carbon nanomaterials for OER.

## 1. Introduction

In recent years, research efforts have increased to solve global energy requirements and improve the alternatives to minimize the oil-derived energy production impact on nature [1,2,3]. New green and sustainable approaches have been proposed to reduce the global contamination impact [4,5]. Among them, energy production based on hydrogen, known as hydrogen economy, has been positioned as the greenest alternative for solving the environmental impacts of fossil fuel emissions [6]. Fuel cell and water splitting systems—in which the oxygen reduction (ORR), oxygen evolution (OER), and (HER) hydrogen evolution reactions play a paramount role—constitute a remarkable example [7,8]. The development of sustainable electrocatalytic materials to carry out these reactions with low onset potential values and high efficiencies is a highly desirable target [9]. For these reasons, research endeavors have been focused on solving individual reaction issues, mainly due to their large dependency on electrocatalyst performance [10]. Currently, high-efficiency activity has been delivered by precious-metal-based nanomaterials [11,12]. Nonetheless, they are not abundant in earth and possess a high cost of production, which makes them unaffordable for widespread applications [13,14,15]. Transition metals—such as Mn, Fe, Ni, Cu, and Co, which are by far less expensive—have been used as starting materials to build non-noble metal-based electrocatalysts with outstanding catalytic performance [16,17]. Cobalt is one of the most abundant and affordable transition metals. It has attracted the attention of the scientific community, since sulfides, phosphides, and hydro(oxi)oxides Co-based compounds have shown competitive results for water-splitting in comparison with precious metals [18,19,20,21,22]. Carbon-based materials have been widely studied for OER, ORR, and HER [23,24]. Waste-derived materials, especially carbonaceous samples [25], have attracted increasing attention due to their great potential and environmentally friendly nature [26]. To develop new classes of green, sustainable, and low-cost carbon-based catalysts, new approaches have emerged [27]. Some strategies have been proposed using biomass or waste-derived materials for both the production of green and sustainable carbonaceous materials with high surface area and for the enhancement of dispersion and exposure of the catalytic sites [28]. Metal- and N-doped carbons have demonstrated to be excellent electrocatalysts [29,30,31]. For instance, nanocarbons such as carbon nanotubes, graphene, borocarbonitride, and covalent triazine framework have been modified with non-metallic atoms, such as N, to support metal nanoparticles, giving rise to ultrahigh catalytic active sites [32,33,34]. The addition of N into the carbon network creates a myriad of structural defects, which decrease the uphill energy states of the catalytic intermediates’ species, thus benefiting the overall ORR activity [35]. Among all the aforementioned approaches, self-doping material represents a good alternative for simplifying synthesis and reducing carbon footprint [36]. Poly-acrylonitrile (PAN) fibers are one of the most used polymers in the textile industry, and they are the principal source of carbon fibers [37,38,39]. To obtain carbon fibers from PAN before carbonization, there are previous steps in which the fibers are cyclized by the nitrile group and finally turned into a self-doped N-carbon, thus improving their conductivity properties [40,41,42,43].

In this work, we propose the modification of post-consumed PAN fibers by mechanochemical-assisted Co addition to obtain Co/N-doped carbon in a solvent-free process. The samples were thermally treated to obtain both Co_3_O_4_ and PAN cyclization, in which the mechanical influence promoted the fiber separation and hence favored a better Co active sites dispersion. Both the effect of temperature and the influence of atmosphere (i.e., air or N_2_) were tested at different Co loadings, improving the synergy between the cyclization and Co_3_O_4_ formation. It was demonstrated that this approach is an efficient method for obtaining sustainable electrocatalysts for energy applications. Finally, the samples delivered suitable electrocatalytic performances for OER in alkaline media, therefore representing a promising alternative for reducing wasted post-consumed PAN fibers.

## 2. Experimental Section

### 2.1. Chemical Reagents

PAN fibers, called acrylic fibers, were obtained on the market, and Co(NO_3_)_2_·3H_2_O (99.5% purity) was acquired from Merck. All the chemicals were used without any further purification steps.

### 2.2. Synthesis of Co-N-Doped Carbon Catalyst

The PAN fibers were cut in small pieces of fibers of around 1 cm, mixed with different loadings of Co(NO_3_)_2_·H_2_O (1, 3, 5, 7, and 10% wt.), and then put into ball mill equipment, employing 10 iron balls (1 cm of diameters) for 30 min at 900 rpm. Subsequently, the samples were calcined at 400 °C for 1 h under air (PAN-A) and N_2_ (PAN-N) atmospheres.

### 2.3. Material Characterization

The structural characterization of the samples was carried out by employing an X-ray diffractometer—a Bruker D8 Discover with Cu Ka radiation. XPS measurements were carried out in an ultrahigh vacuum (UHV) multipurpose surface analysis system, Specs, equipped with a Phoibos 150-MCD energy detector. The experiments were performed at pressures <10^−10^ mbar in “stop and go” mode using an X-ray source. XPS data analysis and quantification was accomplished with CASA software, taking the C1s region as reference for calibration.

The textural studies were conducted using a Micromeritics ASAP 2000 porosimeter instrument (Micromeritics, USA). SEM-EDX micrographs were acquired with a JEOL-SEM JSM-7800 LV scanning microscope (Tokyo, Japan). In addition, TEM images were recorded in a JOEL JEM 1400 instrument and assembled with a charge-coupled camera device (Tokyo, Japan).

### 2.4. Electrochemical Measurements

A water dispersion of 5 mg/mL of sample concentration was prepared and subsequently sonicated for 15 min. Then, a drop of 25 µL was casted on the surface of a 2D glassy carbon disk (5 mm diameter) that was used as a working electrode. After drying overnight, the sample was ready for studying the OER electrocatalyst performance. The latter analyses were performed by linear-sweep voltammetric (LSV) measurements using a classical three-electrode electrochemical cell connected to a Potentiostat/Galvanostat (EmStat 3, PalmSens). Ag/AgCl and graphite rods were used as reference and counter electrodes, respectively. A 0.5 M KOH aqueous solution was employed as an electrolyte. Room temperature electrochemical measurements were recorded in the potential range of 0.00–0.90 V vs. Ag/AgCl, with a scan rate of 2 mV/s and a rotation rate of 1600 rpm. Later, the potentials were referenced to reversible hydrogen electrode (RHE) according to the Nernst equation (E_RHE_ = E_Ag/AgCl_ + 0.059 pH + 0.1976 V) [36,44,45].

An analysis of the number of active sites was also measured, which is proportional to the electrocatalytic surface area (ECSA) [36,46]. As is typical, several cyclic voltammetries (CVs) were measured in a narrow potential window of −0.717 V to −0.817 V vs. RHE (i.e., where no faradaic reactions occurred) at different scan rates (12–48 mV s^−1^ at an interval of 4 mV s^−1^). Sequentially, the slope of the resulting line (areal capacitance) for plotting the scan rate (axe X) against the J anodic-J cathodic (at −0.767 V vs. RHE -axe Y-) was proportional to the ECSA. In addition, electrochemical impedance spectroscopy (EIS) studies were performed to analyze the charge-transfer resistance (R_ct_) of the different samples, aiming to identify the most efficient charge transport (i.e., lower R_ct_ value).

## 3. Results and Discussion

**Figure 1:**
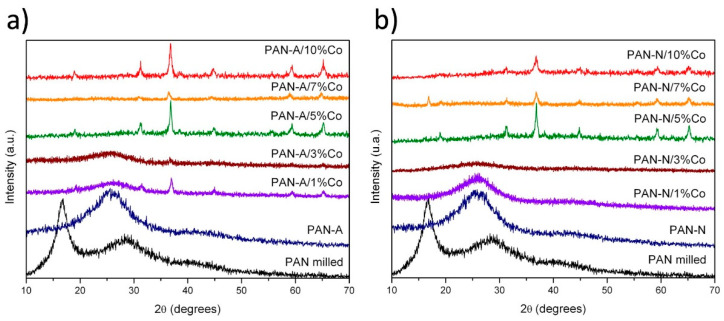
XRD patterns of Co-N-carbon at different Co loading and thermal treatment under air (**a**) and N_2_ (**b**) atmospheres, respectively.

The as-synthesized materials were studied to unveil their structural characteristics using XRD measurements. The diffraction patterns for both atmospheres, air or N_2_, and at different Co loadings are shown in Figure 1a,b, respectively. It is well known that PAN fibers have a semi-crystalline structure [38] due to the presence of an ordered lamellar phase in the PAN molecule. Such structure could be identified by the presence of a principal peak around 17°, even when PAN was mechanochemically treated. Nevertheless, the amorphous structure contribution can be noticed by the appearance of a broad band around 10–35°, most likely due to the bad arrangement of macromolecule chains due to the effect of the milling and the temperature. In addition, when PAN was milled and treated at 400 °C under air or N_2_ atmosphere, the structural arrangement was affected. Remarkably, the crystalline structure was disrupted, leaving only the amorphous phase, which can be associated with the ladder PAN structure by the cyclization of PAN or the crosslinking network promotion. [41] In other words, the crystallinity was lost by the cyclization of the nitrile group in the PAN structure, and a ladder structure was created, which was characterized to be amorphous [46]. This effect was also observed for the 1% and 3% of Co loadings in both atmospheres (air and N_2_). The amorphous PAN underwent an arrangement most likely due to both the Co loading and the thermal treatment modifications. Co entities were mechanochemically dispersed into the PAN fibers, which at higher loading contents disrupted the PAN arrangement, as confirmed by the disappearance of the amorphous contribution. In addition, the thermal treatment allowed the formation of Co_3_O_4_; both events resulted in the complete disruption of the PAN structure, as can be seen in Figure 1a,b for all Co loadings. The Co_3_O_4_ cubic phase was identified by diffraction patterns that matched perfectly with PDF 42-1467 (space group 227/Fd3m). The thermal treatment also had an influence on the formation of Co_3_O_4_. Under air atmosphere (Figure 1a), the principal peaks could be observed from 1% of Co content, while under N_2_ atmosphere (Figure 1b) the main peaks could be visualized just after modification with 5% of cobalt species. Co_3_O_4_ particles deposition into the PAN network resulted in the clear appearance of new peaks associated with the cobalt entities, together with the disappearance of the PAN and milled PAN characteristic signals. Besides, the Co_3_O_4_ intensity peaks could be partially or completely diminished, which could most likely be attributed to an agglomeration or a good dispersion [47].

The chemical features and elemental composition on the surface of the samples were examined by XPS analysis for representative materials, namely, PAN-A, PAN-N, PAN-N/3%Co; PAN-A/3%Co; PAN-N/10%Co; and PAN-A/10%Co. The presence of carbon, nitrogen, and oxygen was detected for all the investigated samples, while in the metal modified materials the appearance of a Co2p corresponding signal clearly assured the successful incorporation of Co entities. The C1s XPS region for PAN-A, PAN-N, PAN-N/3%Co, and PAN-A/3%Co samples displayed four contributions around 284.0 ± 0.5, 286.0 ± 0.5, 288.0 ± 0.5, and 290.0 ± 0.5 eV associated with aromatic and/or graphitic C–C, C–N/C–OH, C=O, and CO_3_^2−^ moieties, respectively. [25] In turn, the samples functionalized with 10% of cobalt exhibited a more striking C–C contribution, together with the non-presence of signals around 290 eV, most likely related to the formation and higher concentration of metal oxide entities on the materials’ surface. Moreover, deconvolution of the N1s XPS region of PAN-A, PAN-N, PAN-N/3%Co, and PAN-A/3%Co samples revealed the presence of five signals located around 397.0 ± 0.5, 399.0 ± 0.5, 400.0 ± 0.5, 402.0 ± 0.5, and 403 ± 0.5 eV, which could be associated with pyridinic-N, pyrrolic-N, amine-N, graphitic-N, and pyridone-N groups [43]. Moreover, as reported by Thomas Wagberg and recently described by Van Der Voort et al., two different types of graphitic nitrogen entities could be contributing to the signal located at 401.0 ± 0.5 eV, namely, the quaternary N atoms at the center (at lower binding energy) and at the valley (at higher binding energy) [35,48].

**Figure 2:**
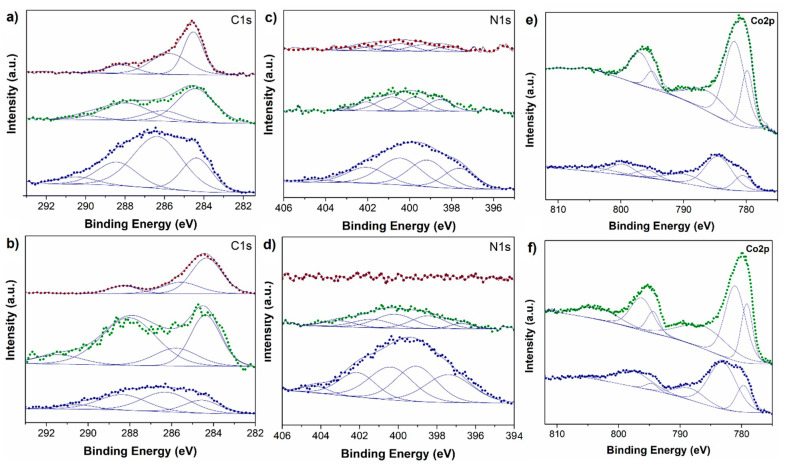
XPS spectra of representative polyacrylonitrile-based Co-N-doped carbon samples prepared under air atmosphere (PAN-A) in the (**a**): C1s, (**c**): N 1s, and (**e**): Co2p regions. XPS spectra of representative polyacrylonitrile-based Co-N-doped carbon samples prepared under N_2_ atmosphere (PAN-N) in the (**b**): C1s, (**d**): N 1s, and (**f**): Co2p regions. Blue line: unmodified PAN based materials; green line: 3% Co modified materials; red line: 10% Co modified materials.

In addition, for higher cobalt content in the samples (10% of Co), the nitrogen signal was practically negligible, which could most likely be associated with an almost full coverage of the nitrogen-containing surface of PAN by cobalt oxide species. Furthermore, Figure 2e,f show the clear appearance of a Co2p signal for the metal-modified materials, with the concomitant presence of Co2p_3/2_ and Co2p_1/2_ peaks of Co (II) and Co (III) species, confirming the formation of Co_3_O_4_ entities. In addition, satellite peaks were also observed at relatively lower intensity [22,36].

Quantification analysis based on XPS data was carried out as shown in Table 1. It is worth highlighting that for 3%Co-PAN-N, the highest Co/C%At. concentration ratio on the surface was found, and at the same time for cobalt modified samples, this material exhibited the highest nitrogen content, which could be further related to the electrocatalytic behavior. As previously mentioned, nitrogen contents lower than 2%At. concentration on the surface of the materials were found for 10%Co-PAN-N and 10%Co-PAN-A.

The textural properties of Co-modified N-doped carbonaceous samples were analyzed by N_2_ adsorption and desorption isotherms, and the most representative samples are shown in Table 2. Surface area values were obtained for Co modified N-doped carbon samples, and it was found that although there was not a clear trend for different Co loadings, the metallic particles significantly varied the textural characteristics of the resulting nanomaterials. The different Co loadings in PAN could have risen up to 34.87 m^2^/g (more than twice) or decreased to 1.79 m^2^/g the S_BET_, in comparison with PAN milled and heated under N_2_ atmosphere. Similar effects occurred with the pore volume, which varied from 11 to 65 nm. In addition, the synthetic atmosphere did not show a clear effect, but in any case, under N_2_ atmosphere, high Co loadings gave rise to a maximum S_BET_ area of 34.87 m^2^/g. These results are similar to those reported in the literature for PAN [43] and g-Co_3_O_4_/g-C_3_N_4_ materials [36].

**Figure 3:**
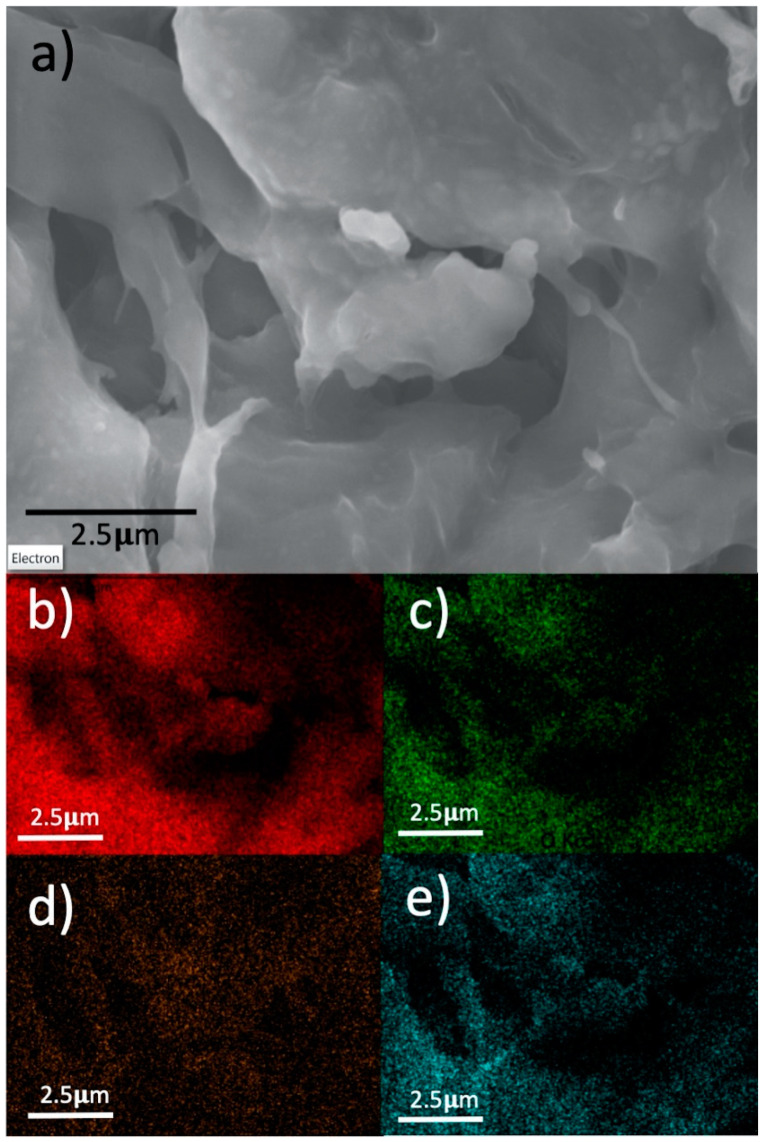
Representative SEM image of PAN-N/3%Co sample (**a**), and its corresponding SEM-EDX mapping for the following elements: carbon (**b**), nitrogen (**c**), cobalt (**d**), and Oxygen (**e**).

The morphology of the samples was studied by SEM images, and a representative sample is shown in the Figure 3a. Additionally, an SEM-EDX mapping analysis was performed to display the superficial atomic content distribution (see Supplementary Materials, Figures S1 and S2). As can be seen in Figure 3b–e, carbon, nitrogen, cobalt, and oxygen contents were homogeneously distributed in all the Co modified N-doped carbon samples. It was clearly observed that nitrogen and cobalt atoms were located inside of the carbon structure. This analysis confirmed that the mechanochemical approach is an easy way to obtain homogeneous materials with well dispersed Co species.

**Figure 4:**
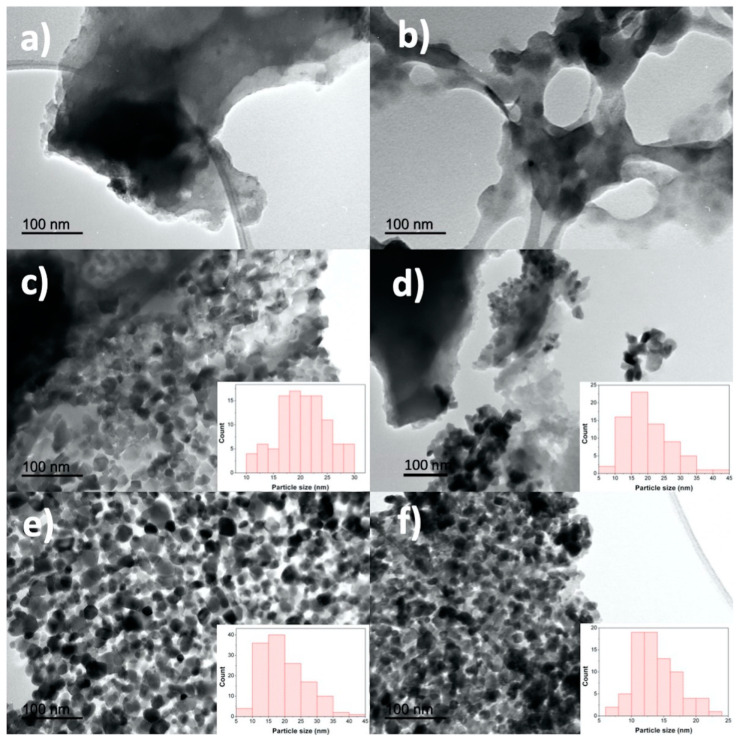
TEM images for the representative samples (**a**) PAN-A, (**b**) PAN-N, (**c**)PAN-A/3%Co, (**d**) PAN-N/3%Co, (**e**) PAN-A/10%Co and (**f**) PAN-N/10%Co. The inset (**c**–**f**) shows the particles size distribution.

In addition, the Co-N-doped carbon materials were also studied by TEM images. Representative samples are shown in the Figure 4. The N-Co-doped carbon morphology was exposed to both atmospheres, in which we can appreciate the morphology effect by air (Figure 4a,c,e) and N_2_ (Figure 4b,d,f). It is worth noting that the morphology was different under air and N_2_ atmospheres, appreciating that the samples showed similar lamellar or reticular structure, respectively. In any case, afterward the Co loadings of the structures were interrupted by the Co nanoparticles’ agglomerations under both atmospheres; whereas under air atmosphere, it could be observed (Figure 4c,e) that nanoparticles were better defined than those obtained under N_2_, which could be associated with the oxygen deficiency to promote the Co_3_O_4_ phase. Additionally, it is notable that for the sample PAN-A/3%Co (Figure 4c), the nanoparticles were more angular than the PAN-A/10%Co nanoparticles (Figure 4e), which were more circular in shape. Also, both samples showed a uniform size distribution (Figure 4c,e insets). In addition, the sample PAN-N/3%Co (Figure 3d) showed that the N_2_ atmosphere reduced the Co_3_O_4_ nanoparticles agglomeration, in which the size distribution was gradually reduced and well dispersed compared to the PAN-A/3%Co (Figure 4c inset). Furthermore, the maximum Co loading favored Co_3_O_4_ nanoparticles agglomeration in both atmospheres, but in N_2_ the particle size (13 nm) was relatively lower than the size obtained under air (18 nm), which can explain why PAN-N/10%Co (34.87 m^2^/g) had higher S_BET_ than the PAN-A/10%Co sample (25.72 m^2^/g) (see Table 2).

### OER Electrochemical Analysis

**Figure 5:**
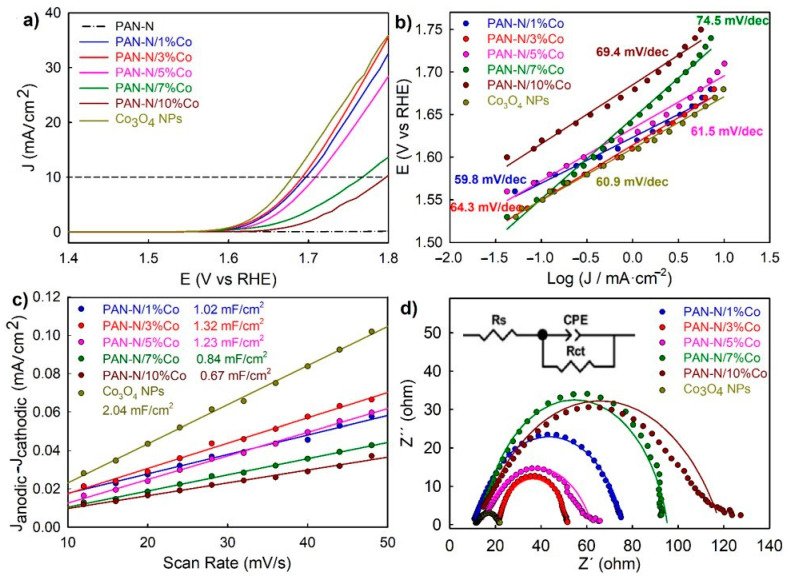
(**a**) Oxygen evolution reactions (OER) polarization curves obtained for the different PAN-N/Co samples, including the reference (Co_3_O_2_ NPs), in 0.5 M KOH solution at 2 mV/s; (**b**) Tafel plots of the samples. The values are the slope of each curve: (**c**) Plots of difference of anodic and cathodic current density as a function of scan rate and (**d**) electrochemical impedance spectroscopy (EIS) obtained for the samples at the overpotential of 700 mV, with frequency from 100 kHz to 1 Hz and amplitude of 5 mV. Dots and lines represent the experimental and simulated data, respectively.

The OER electrocatalytic performance for the different synthesized cobalt-doped PAN-N samples were investigated through LSV curves conducted using a three-electrode setup in 0.5 M KOH electrolyte. Figure 5a shows that the resulting current density increased with the potential change for the different samples, which is expected for OER anodic processes. For the evaluation of their electrocatalyst activity, the overpotentials at the geometric current density of 10 mA/cm^2^ were measured [36,49]. As shown in Figure 5a, the PAN-N/3%Co showed the lowest overpotential of 460 mV at 10 mA/cm^2^, which was quite similar to the one obtained with the reference material (i.e., Co_3_O_4_ NPs, 450 mV). Table S1 compared the resulting OER electrocatalytic performance against other Co@carbon-based OER catalysts reported. As expected, in agreement with the previous characterization, the increase of the Co loading did not improve the overpotential of PAN-N/3%Co, even producing the opposite effect (e.g., 480 mV for PAN-N/5%Co, 530 mV for PAN-N/7%Co and 560 mV PAN-N/10%Co). This fact can be attributed to the limited number of Co-N-C available sites for OER on our sustainable support material, which can be considered as OER active sites because it is the reverse reaction of the ORR [50,51]. It is worth noting that the presence of reduced amounts of Co on the PAN-N network would benefit the uniform distribution of single atom and/or tiny metallic clusters, thus improving both the number of accessible OER catalytic sites and the overall electrocatalytic rates. In addition, the Tafel slope was determined to analyse the mechanism and kinetics of the OER rate-determining step (Figure 5b). All the resulting OER Tafel slopes had similar values, which were close to 60 mV/dec, exhibiting a comparable Tafel slope to Pt under alkaline conditions [52,53,54,55]. These findings imply that the Co-doped PAN-N materials promoted ultrahigh OER kinetics; in general, the low Co content samples were the ones with the lower Tafel slopes. These low Tafel slope values indicate that the second reaction of the OER mechanism in alkaline media was rate-determining [56]. The low Tafel slope also indicates a strong adsorption of surface intermediates in the primary step, hampering the following step which became rate limiting [57]. In summary, the different Co-doped PAN-N samples showed similar surface adsorption energies, being almost independent of the distinct Co loading.

The total electrode activity was determined by the total number of active sites [36,46]. Figure 5c shows the plots of the difference of anodic and cathodic current density versus the scan rate, in which the slope of the curves (areal capacitance) was proportional to their ECSA. PAN-N/3%Co showed the highest areal capacitance of 1.32 mF/cm^2^, which was close to the one obtained for the reference Co_3_O_4_ NPs of 2.04 mF/cm^2^. These results suggest that PAN-N/3%Co had the highest number of active sites among the different Co-doped PAN-N samples, which strongly supports our previous explanations. Furthermore, the higher number of active sites was favorable for charge transfer. Electrochemical impedance spectroscopy (EIS) studies were conducted for the different cobalt-doped PAN-N samples and the reference (Figure 5d). The EIS results were fitted based on the equivalent circuit (Figure 5d, inset), and the obtained solution resistance (R_s_) and charge-transfer resistance (R_ct_) are summarized in Table 3 [58]. As expected, PAN-N/3%Co had the smallest R_ct_ of 152.55 Ω/cm^2^, while PAN-N/10%Co had the highest R_ct_ of 528.00 Ω/cm^2^. It is also noteworthy that the increase of cobalt loadings into PAN-N affects its charge transport efficiency.

Finally, a long-term stability study was performed with the PAN-N/3%Co sample by chronopotentiometry at a current density of 10 mA·cm^−2^ for 12 h [22,36]. As it can be observed in Figure S4, the overpotential remained almost constant during this durability test, demonstrating the good electrochemical stability of this sample.

## 4. Conclusions

We reported a new class of Co-N-doped carbon, mechanochemically synthetized by using a solvent-free process as a new ecofriendly use of post-consumed PAN fibers. The Co-N-doped carbon materials exhibited better catalytic performance when the materials had been synthetized under N_2_ atmosphere. The Co loading had a significant impact on the catalytic OER performance in an alkaline environment. The PAN-N/3%Co nanocomposite revealed the highest active sites distribution, with the enhanced charge transport efficiency exhibiting a small onset potential of 460 mV at a current of 10 mA/cm^2^. The design of a green route and environmentally friendly strategy to make Co-N-doped carbon from PAN post-consumed fibers is a great alternative for fabricating low-cost catalytic systems for OER.

## Figures and Tables

**Table 1:** Elemental composition of the prepared materials calculated from the XPS data.

Sample	C1s (%At. Conc.)	O1s (%At. Conc.)	N1s (%At. Conc.)	Co2p (%At. Conc.)	Co/C Ratio
PAN-A	35.5	28.2	36.3	ND.	
PAN-N	30.3	25.9	43.8	ND.	
3%Co-PAN-A	16.5	52.2	7.9	23.4	1.4
3%Co-PAN-N	11.8	53.2	10.2	24.8	2.1
10%Co-PAN-A	14.2	58.2	1.8	25.8	1.8
10%Co-PAN-N	13.8	59.3	0.7	26.2	1.9

ND. Not detected.

**Table 2:** Textural properties obtained for PAN and Co-N-doped carbon representative materials obtained by nitrogen physisorption measurements.

Catalyst	S_BET_^[a]^ (m^2^/g)	V_BJH_^[b]^ (cm^3^/g)	D_BJH_^[c]^ (nm)
PAN milled	12.53	0.012	65
PAN-N	16.48	0.014	63
PAN-A/3%Co	8.79	0.025	11
PAN-N/3%Co	1.79	0.003	15
PAN-A/10%Co	25.72	0.089	13
PAN-N/10%Co	34.87	0.163	23

^[a]^ SBET: Specific surface area was calculated by the Brunauer–Emmet-Teller (BET) equation. ^[b]^ VBJH: Mean pore volumes were calculated by the Barret–Joyner–Halenda (BJH) equation. ^[c]^ DBJH: Mean pore size diameter was calculated by the Barret–Joyner–Halenda (BJH) equation.

**Table 3:** Resulting data from the simulated equivalent circuit for the different Co-doped PAN-N samples and the reference Co_3_O_4_ NPs. Data values reported for other Co@NC-based electrocatalysts are included.

Sample	R_s_ [Ω/cm^2^]	R_ct_ [Ω/cm^2^]	CPE-T	CPE-P
PAN-N/1%Co	53.85	326.50	6.00 × 10^−5^	0.7798
PAN-N/3%Co	106.55	152.55	1.10 × 10^−4^	0.8490
PAN-N/5%Co	70.10	241.50	4.15 × 10^−5^	0.7000
PAN-N/7%Co	70.65	406.00	1.40 × 10^−5^	0.8604
PAN-N/10%Co	64.65	528.00	8.00 × 10^−6^	0.6979
Co_3_O_2_ NPs	58.20	52.35	6.50 × 10^−4^	0.6905
Co-NC [59]	-	108.2	-	-
Fe-Co-NCNFs-700 [60]	-	57.25	-	-

## Data Availability

The data presented in this study are available on request from the corresponding author.

## Supplementary Materials

The following are available online at https://www.mdpi.com/2079-4991/11/2/290/s1: Figure S1. (a) SEM-EDX for PAN-N (0% Co) and their elemental mapping of (b) carbon, (c) nitrogen, (d) oxygen and (e) Iron, no detection, we can discard the impurity by iron ball milling, Figure S2. (a) SEM-EDX for PAN-AIR/3%Co and their elemental mapping of (b) carbon, (c) nitrogen, (d) cobalt and (e) oxygen, Figure S3. OER polarization curves obtained for the different PAN-AIR/Co samples, including the reference (Co_3_O_2_ NPs) in 0.5 M KOH solution at 2 mV/s, Figure S4. Chronopotentiometry measurements at J = 10 mA/cm2 for the PAN-N/3%Co sample, Table S1. A comparison on the OER electrocatalytic activity of our PAN-N/3%Co sample and other reported Co@Carbon-based OER catalysts.
