# Supplementary material for: Mechanochemically Synthetized PAN-Based Co-N-Doped Carbon Materials as Electrocatalyst for Oxygen Evolution Reaction
Source: Nanomaterials (Basel). 2021 Jan 22;11(2):290. doi: 10.3390/nano11020290 (PMC7911492; doi:10.3390/nano11020290)
Supplement: Supplementary file 1 [file nanomaterials-11-00290-s001.pdf]

nanomaterials

MDPI

Article

Mechanochemically Synthetized PAN-Based Co-N-Doped Carbon Materials as Electrocatalyst for Oxygen Evolution Reaction

Paulette Gómez-López [1], José Ángel Salatti-Dorado [2], Daily Rodríguez-Padrón [1], Manuel Cano [2], Clemente G. Alvarado-Beltrán [3], Alain R. Puente Santiago [4], Juan J. Giner-Casares [2,*] and Rafael Luque [1,5,*]

1 Grupo FQM-383, Departamento de Química Orgánica, Universidad de Córdoba, E-14071 Córdoba, Spain; z82golop@uco.es (P.G.-L.); dailydggs@gmail.com (D.R.-P.)
2 Departamento de Química Física y Termodinámica Aplicada, Instituto Universitario de Nanoquímica (IUNAN), Facultad de Ciencias, Universidad de Córdoba, Campus de Rabanales, Ed. Marie Curie, E-14071 Córdoba, Spain; a72sadoj@uco.es (J.Á.S.-D.); q82calum@uco.es (M.C.)
3 Facultad de Ingeniería Mochis, Universidad Autónoma de Sinaloa, Fuente de Poseidón y Prol. Angel Flores, S.N., 81223 Los Mochis Sin., Mexico; calvarado@uas.edu.mx
4 Department of Chemistry and Biochemistry, University of Texas at El Paso, 500 West University Avenue, El Paso, TX 79968, USA; arpuentesan@utep.edu
5 Scientific Center for Molecular Design and Synthesis of Innovative Compounds for the Medical Industry, People's Friendship University of Russia (RUDN University), 117198 Moscow, Russia
* Correspondence: jjginer@uco.es (J.J.G.-C.); q62alsor@uco.es (R.L.)

Abstract: We report a new class of polyacrylonitrile (PAN)-based Co-N-doped carbon materials that can act as suitable catalyst for oxygen evolution reactions (OER). Different Co loadings were mechanochemically added into post-consumed PAN fibers. Subsequently, the samples were treated at 300 °C under air (PAN-A) or nitrogen (PAN-N) atmosphere to promote simultaneously the Co_3O_4 species and PAN cyclization. The resulting electrocatalysts were fully characterized and analyzed by X-ray diffraction (XRD) and photoelectron spectroscopy (XPS), transmission (TEM) and scanning electron (SEM) microscopies, as well as nitrogen porosimetry. The catalytic performance of the Co-N-doped carbon nanomaterials were tested for OER in alkaline environments. Cobalt-doped PAN-A samples showed worse OER electrocatalytic performance than their homologous PAN-N ones. The PAN-N/3% Co catalyst exhibited the lowest OER overpotential (460 mV) among all the Co-N-doped carbon nanocomposites, reaching 10 mA/cm^2. This work provides in-depth insights on the electrocatalytic performance of metal-doped carbon nanomaterials for OER.

Keywords: mechanochemical synthesis; carbon N-doped; Co_2O_3 nanoparticles; PAN; OER

Citation: Gómez-López, P.; Salatti-Dorado, J.Á.; Rodríguez-Padrón, D.; Cano, M.; Alvarado-Beltrán, C.G.; Puente-Santiago, A.R.; Giner-Casares, J.J.; Luque, R. Mechanochemically Synthetized PAN-Based Co-N-Doped Carbon Materials as Electrocatalyst for Oxygen Evolution Reaction. *Nanomaterials* **2021**, *11*, 290. https://doi.org/10.3390/nano11020290

Received: 21 December 2020
Accepted: 20 January 2021
Published: 22 January 2021

Academic Editor: Gabriele Giancane

Publisher's Note: MDPI stays neutral with regard to jurisdictional claims in published maps and institutional affiliations.

Copyright: © 2021 by the authors. Submitted for possible open access publication under the terms and conditions of the Creative Commons Attribution (CC BY) license (http://creativecommons.org/licenses/by/4.0/).

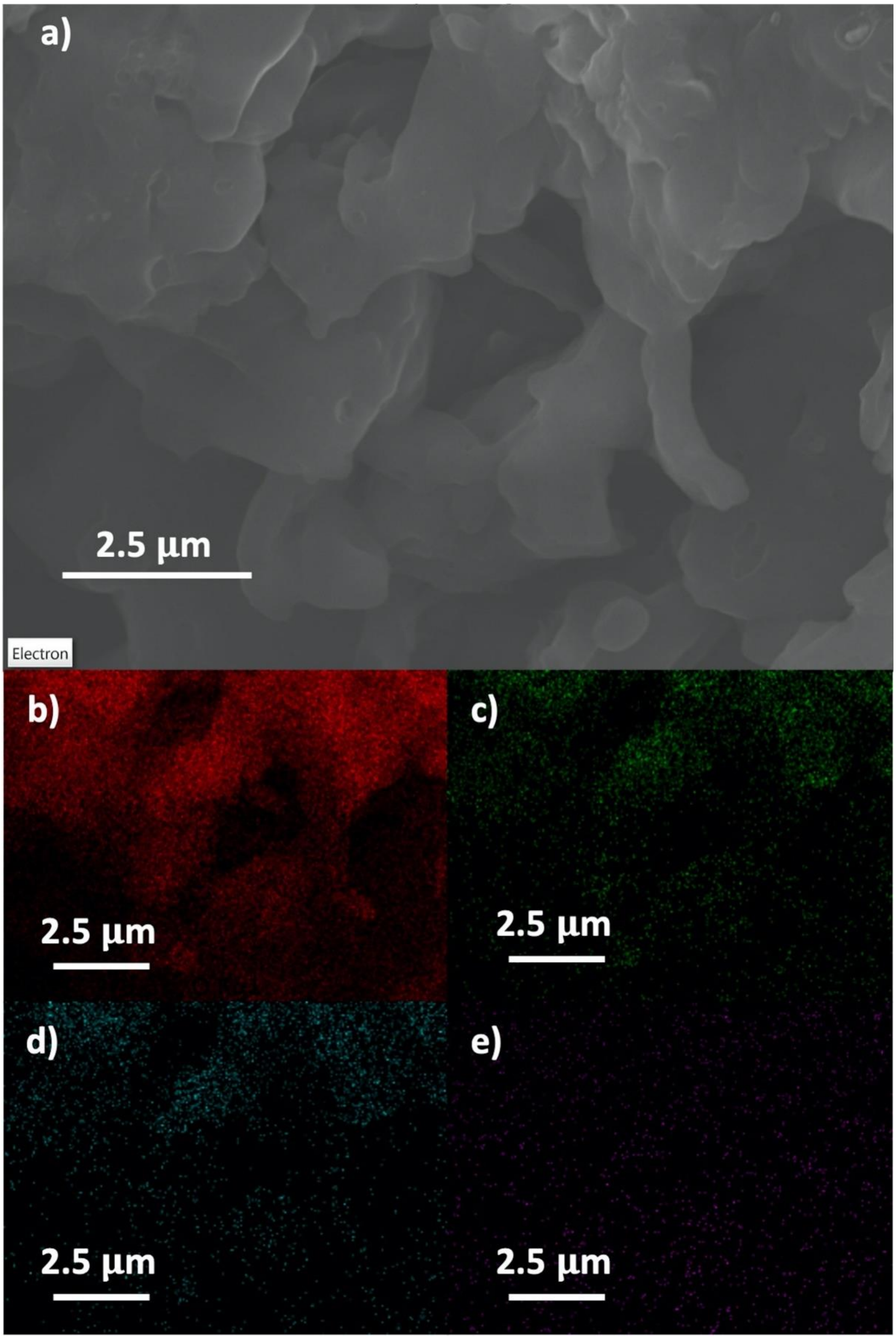

Figure S1. (**a**) SEM-EDX for PAN-N (0% Co) and their elemental mapping of (**b**) carbon, (**c**) nitrogen, (**d**) oxygen and (**e**) Iron, no detection, we can discard the impurity by iron ball milling.

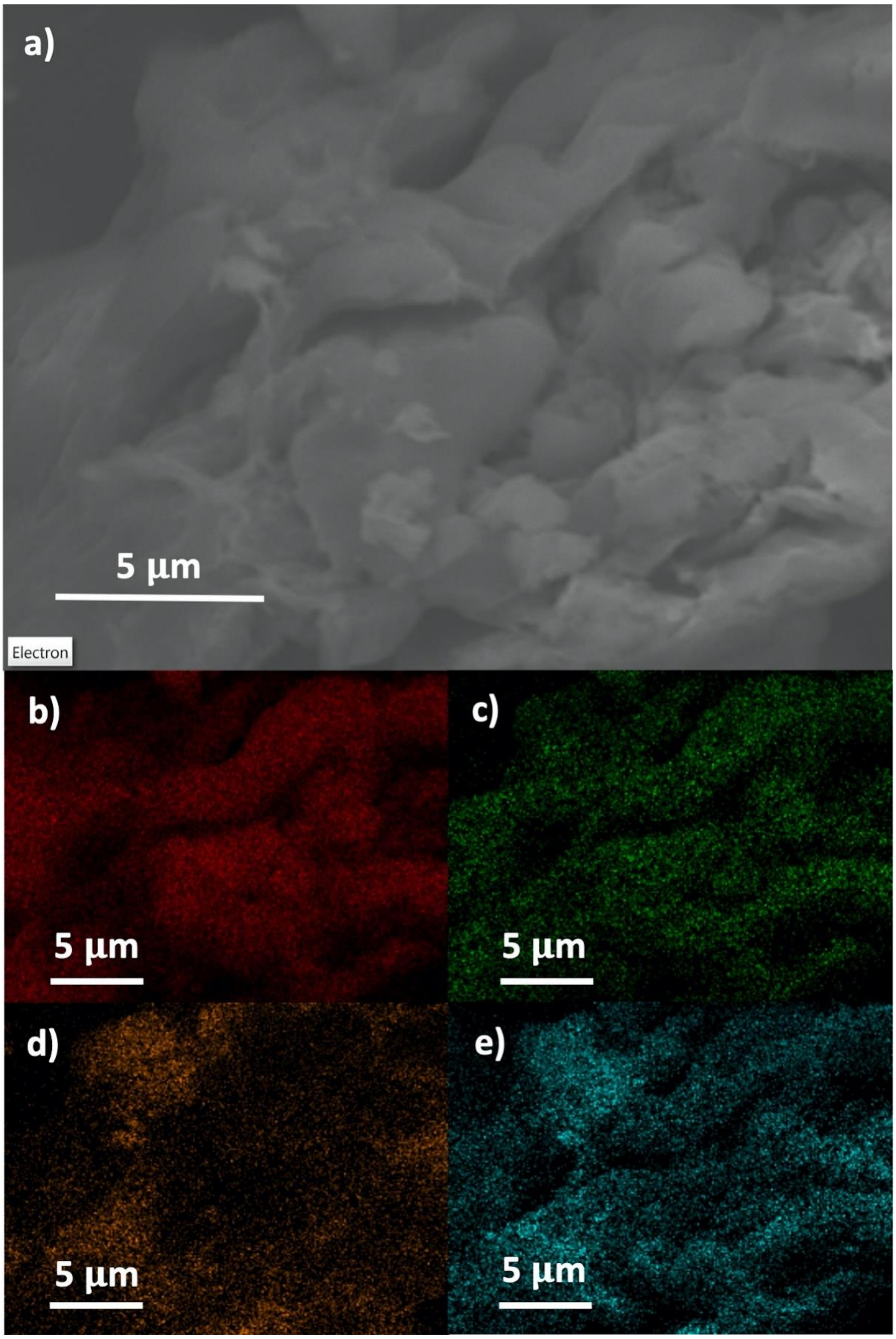

Figure S2. (**a**) SEM-EDX for PAN-AIR/3%Co and their elemental mapping of (**b**) carbon, (**c**) nitrogen, (**d**) cobalt and (**e**) oxygen.

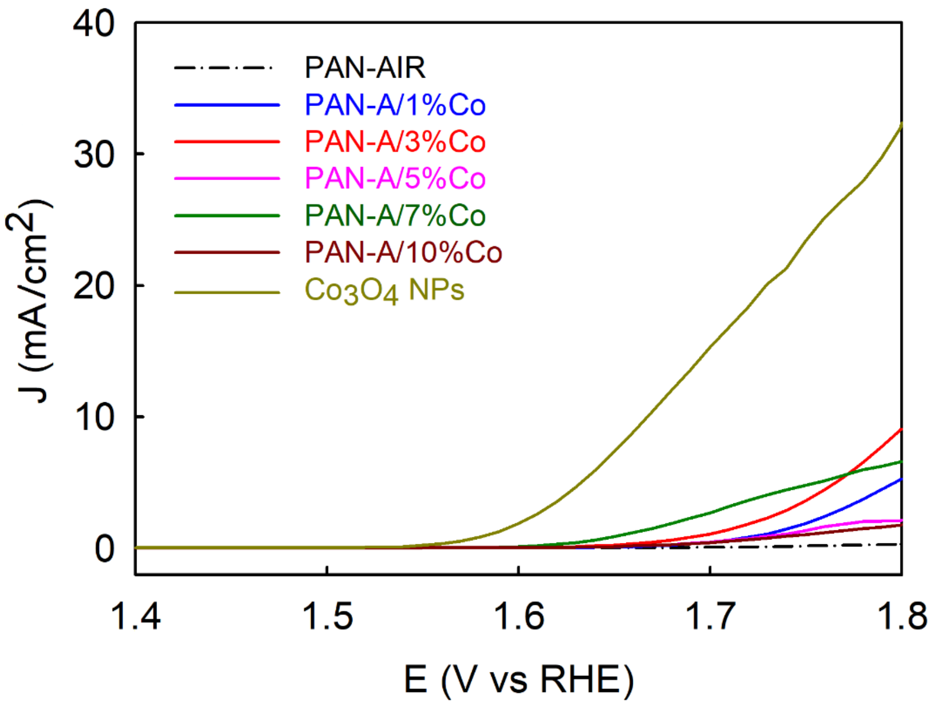

Figure S3. OER polarization curves obtained for the different PAN-AIR/Co samples, including the reference (Co_3O_2 NPs) in 0.5 M KOH solution at 2 mV/s.

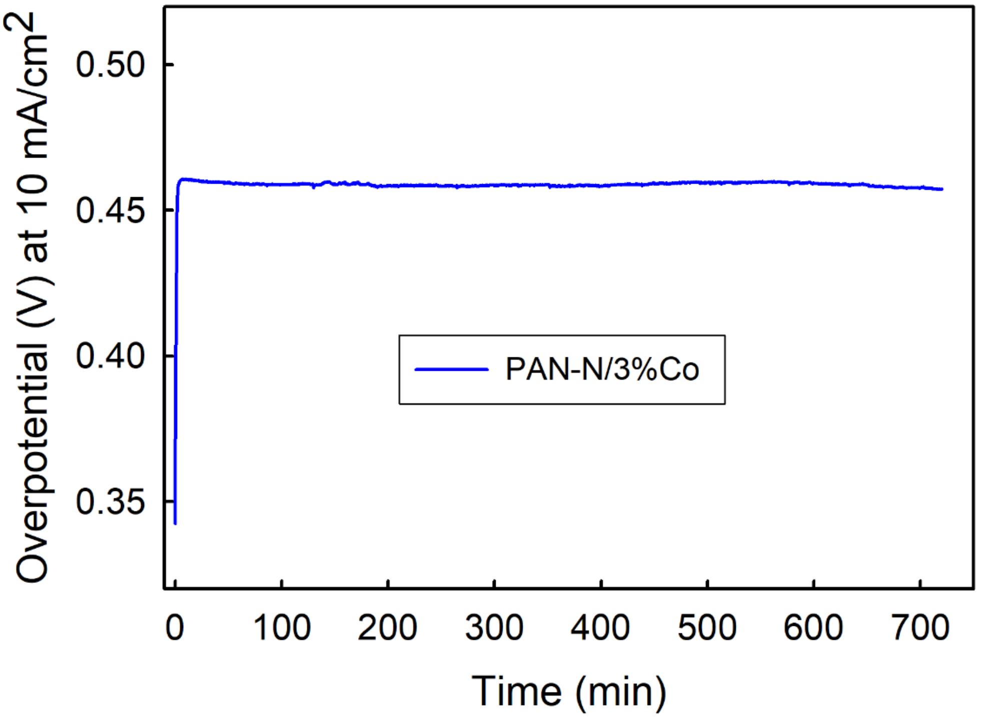

Figure S4. Chronopotentiometry measurements at J = 10 mA/cm^2 for the PAN-N/3%Co sample.

Table S1. A comparison on the OER electrocatalytic activity of our PAN-N/3%Co sample and other reported Co@Carbon-based OER catalysts.

Co@C Based Catalysts	η_{10} (mV)	Tafel slope (mV/dec)	References
Co_9S_8/C nanosheets	434	122	[S1]
CoCMP	340	87	[S2]
Co@C600	277	46	[S3]
Co–B/C	320	75	[S4]
Co/CoO-C	340	84.5	[S5]
Co/CoO_x@BS-NCNTs-700	360	112	[S6]
CoC_x/FeCo@C/rGO	390	99.1	[S7]
MoS_2/Co–N–CN_2	410	74	[S8]
Co–Ni–P/MoS	300	71	[S9]
Co_3S_4@$NiMoO_4$	320	102	[S10]
Co-Mo_2C@NGCS-2	360	54	[S11]
$ACTP_2$@Co,N-800	374	98	[S12]
$Co_2N_{0.67}$-BHPC	340	90	[S13]
Co/N-CNT (1500 SCCM)	310	66	[S14]
PBA-Pd-Co/C	500		[S15]
PAN-N/3%Co	460	60	This work

References

[S1] Li, L.; Song, L.; Guo, H.; Xia, W.; Jiang, C.; Gao, B.; Wu, C.; Wang, T.; He, J. N-Doped porous carbon nanosheets decorated with graphitized carbon layer encapsulated Co_9S_8 nanoparticles: an efficient bifunctional electrocatalyst for the OER and ORR. *Nanoscale* **2019**, *11*, 901-907.

[S2] Singh, A.; Roy, S.; Das, C.; Samanta, D.; Maji, T. K. Metallophthalocyanine-based redox active metal-organic conjugated microporous polymers for OER catalysis. *Chem. Commun.* **2018**, *54*, 4465–4468.

[S3] Li, M.; Bai, L.; Wu, S.; Wen, X.; Guan, J. Co/CoOx Nanoparticles Embedded on Carbon for Efficient Catalysis of Oxygen Evolution and Oxygen Reduction Reactions. *ChemSusChem* **2018**, *11*, 1722–1727.

[S4] Li, Y.; Xu, H.; Huang, H.; Zhao, Y.; Ma, T. Synthesis of Co–B in porous carbon using a metal–organic framework (MOF) precursor: A highly efficient catalyst for the oxygen evolution reaction. *Electrochem. Commun.* **2018**, *86*, 140–144.

[S5] Lu, H.-S.; Zhang, H.; Zhang, X.; Sun, N.; Zhu, X.; Zhao, H.; Wang, G. Transformation of carbon-encapsulated metallic Co into ultrafine Co/CoO nanoparticles exposed on N-doped graphitic carbon for high-performance rechargeable zinc-air battery. *Appl. Surf. Sci.* **2018**, *448*, 369–379.

[S6] Zhang, P.; Cai, Z.; You, S.; Wang, F.; Dai, Y.; Zhang, C.; Zhang, Y.; Ren, N.; Zou, J. Self-generated carbon nanotubes for protecting active sites on bifunctional Co/CoO_x schottky junctions to promote oxygen reduction/evolution reactions via efficient valence transition. *J. Colloid Interf. Sci.* **2019**, *557*, 580–590.

[S7] Fang, H.; Huang, T.; Sun, Y.; Kang, B.; Liang, D.; Yao, S.; Yu, J.; Dinesh, M. M.; Wu, S.; Lee, J. Y.; Mao, S. Metal-organic framework-derived core-shell-structured nitrogen-doped CoC_x/FeCo@C hybrid supported by reduced graphene oxide sheets as high performance bifunctional electrocatalysts for ORR and OER. *J. Catal.* **2019**, *371*, 185–195.

[S8] Hou, X.; Zhou, H.; Zhao, M.; Cai, Y.; Wei, Q. MoS_2 Nanoplates Embedded in Co−N-Doped Carbon Nanocages as Efficient Catalyst for HER and OER. *ACS Sustainable Chem. Eng.* **2020**, *8*, 5724−5733.

[S9] Bao, J.; Zhou, Y.; Zhang, Y.; Sheng, X.; Wang, Y.; Liang, S.; Guo, C.; Yang, W.; Zhuang, T.; Hu, Y. Engineering water splitting sites in three-dimensional flower-like Co–Ni–P/MoS_2 heterostructural hybrid spheres for accelerating electrocatalytic oxygen and hydrogen evolution. *J. Mater. Chem. A*, **2020**, *8*, 22181–22190.

[S10] Chen, L.; Xu, G. C.; Xu, G.; Zhang, L.; Ding, H. Co-based coordination polymer-derived Co_3S_4 nanotube decorated with $NiMoO_4$ nanosheets for effective oxygen evolution reaction. *Int. J. Hydrog. Energy* **2020**, *45*, 30463−30472.

[S11] Jiang, A.; Zhang, W.; Wang, Z.; Nidamanuri, N. P.; Li, Q.; Zeng, X. C.; Dong, M. Direct synthesis of bifunctional nanorods from a Co–adenine–MoO_3 hybrid for overall water splitting. *Mater. Chem. Front.* **2020**, *4*, 546−554.

[S12] Zhang, J.; Zhang, T.; Ma, J.; Wang, Z.; Liu, J.; Gong, X.ORR and OER of CoeN codoped carbon-based electrocatalysts enhanced by boundary layer oxygen molecules transfer. *Carbon* **2021**, *172*, 556–568.

[S13] Lv, X.; Xiao, Z.; Wang, H.; Wang, X.; Shan, L.; Wang, F.; Wei, C.; Tang, X.; Chen, Y. In situ construction of Co/N/C-based heterojunction on biomass-derived hierarchical porous carbon with stable active sites using a Co-N protective strategy for high-efficiency ORR, OER and HER trifunctional electrocatalysts. *J. Energy Chem.* **2021**, *54*, 626–638.

[S14] Han, H.; Paik, J. W.; Ham, M.; Kim, K. M.; Park, J. K.; Jeong, Y. K. Atomic Layer Deposition-Assisted Fabrication of Co-Nanoparticle/N-Doped Carbon Nanotube Hybrids as Efficient Electrocatalysts for the Oxygen Evolution Reaction. *Small* **2020**, *16*, 2002427.

[S15] Liu, X.; Han, J.; Deng, J.; Imhanria, S.; Ren, Z.; Wang, W. Prussian blue analogue derived Pd-Co composite bifunctional electrocatalyst for Zneair battery. *J. Alloy. Compd.* **2020**, *832*, 154896.
